# Clinical, Endoscopic and Histologic Differences in Gastric Mucosa Between Younger and Older Adults: An Observational Study on the Aging Stomach

**DOI:** 10.3390/medsci13040224

**Published:** 2025-10-08

**Authors:** Francisco Vara-Luiz, Ivo Mendes, Carolina Palma, Paulo Mascarenhas, Ana Elisa Teles, Inês Costa Santos, Gonçalo Nunes, Marta Patita, Irina Mocanu, Sara Pires, Tânia Meira, Ana Vieira, Pedro Pinto-Marques, Daniel Gomes-Pinto, Jorge Fonseca

**Affiliations:** 1 Gastroenterology Department, Hospital Garcia de Orta, 2805-267 Almada, Portugal; 2 Aging Lab, Egas Moniz Center for Interdisciplinary Research (CiiEM), Egas Moniz School of Health and Science, 2829-511 Almada, Portugal; 3 Pathology Department, Hospital Garcia de Orta, 2805-267 Almada, Portugal

**Keywords:** aging, chronic gastritis, intestinal metaplasia, atrophic gastritis, *Helicobacter pylori*

## Abstract

Background/Objectives: Age-related changes in the gastric mucosa remain incompletely understood. We aimed to assess and compare clinical, endoscopic and histologic changes in the gastric mucosa associated with aging, and to explore whether gastric aging is associated with a distinct histological pattern. Methods: Single-center observational study. Younger (18–45 years) and older (≥70 years) adults undergoing elective upper endoscopy were included and underwent gastric biopsies. The clinical, endoscopic and histologic features were analyzed and compared. Results: A total of 100 patients were included (45 men/55 women), 50 with 18–45 years and 50 with ≥70 years. Dyspepsia, gastro-esophageal reflux disease and peptic ulcer disease were the most common indications for upper endoscopy. Gastric lesions (erythema, erosions, ulceration and polyps) were more common in older patients (80% vs. 50%, *p* = 0.003), as well as histologic changes such as chronic gastritis (56% vs. 38%, *p* = 0.004), chronic atrophic gastritis (CAG; 28% vs. 4%, *p* < 0.001) and intestinal metaplasia (28% vs. 4%, *p* < 0.001). These findings persisted after adjusting for *Helicobacter pylori* (*H. pylori*) status and proton pump inhibitor intake on the multivariate analysis. Prevalence of *H. pylori* was similar between both groups (28% vs. 32%, *p* = 0.189). Conclusions: Aging is associated with clinical, endoscopic and histologic changes in the gastric mucosa including CAG and metaplasia, independent of the presence of *H. pylori*. These findings may result from several aging-related pathophysiological processes and decades of cumulative gastric injury and support the hypothesis of an aging stomach phenotype, underscoring the need for an age-adjusted interpretation of gastric biopsies.

## 1. Introduction

Society is witnessing a remarkable increase in life expectancy, resulting in an aging population worldwide [1]. By the year 2050, the global population of individuals aged 60 years and over is predicted to increase from 12% to 22%, with the proportion of those aged 80 and over rising threefold [2]. In the context of this demographic shift, healthcare workers now require a basic understanding of essential aging medicine to provide an evidence-based clinical approach to older patients [3].

Aging is a biological process that causes loss of tissue and decreasing organ function. It is characterized by damage to DNA, telomere shortening, increased production of reactive oxygen species and mitochondrial dysfunction. All organs and systems, including the gastrointestinal tract, are affected by this process of physiological and pathological changes [4]. While some attention has been devoted to age-related changes in colonic motility [5], pancreatic function [6] and hepatic metabolism [7], the stomach has historically received less focus in the context of normal aging. Nonetheless, the stomach plays a role in digestion, nutrient absorption and mucosal defense, and subtle structural changes may carry important clinical implications. However, current knowledge about gastric changes in the older population is scarce, as most clinical trials exclude elderly patients [8].

Clinical and experimental studies in the stomach of older adults have reported (1) impaired gastric mucosal defenses (decrease in bicarbonate, mucus and prostaglandin secretion, as well as reduced nitric oxide synthase activity), (2) reduced gastric blood flow, (3) decreased postprandial peristalsis/gastric contractile force, which may result in a delay of gastric emptying, (4) modification in appetite peptide production (i.e., leptin and ghrelin) and (5) changes in microbial diversity [9,10,11]. Moreover, immunosenescence, resulting in a gradual decline in immune function, can exacerbate the severity of diseases, especially in individuals who are malnourished or otherwise at risk [12,13].

The prevalence of different gastric lesions detected at upper gastrointestinal endoscopy has changed over time [14]. With the progressively older population, over recent years there has been a shift towards factors that may result in changes in the gastric mucosa, such as diet, increased prevalence of obesity, *Helicobacter pylori* (*H. pylori*) infection, although it seems to be decreasing in developed countries [15], and medications that may chronically damage the gastric mucosa [16]. Those are just few examples of aggressive factors that call for an update in the interpretation of endoscopic lesions found in the elderly. Indeed, older patients have a much greater susceptibility of chronic injury to the gastric mucosa than younger patients. Nearly 40% of people over 60 years old experience gastrointestinal issues often linked to the decline in digestive function that accompanies aging [17,18].

Previously, gastritis used to be considered a normal development associated with aging [19], but currently this concept is challenged in the scientific literature. During the aging process, gastric mucosa may undergo cumulative injury and reparative remodeling, potentially resulting in chronic inflammation, glandular atrophy and metaplastic transformation. However, mucosal changes related to gastric morphology and histology in the elderly focus mainly on atrophic changes and intestinal metaplasia (IM) associated with *H. pylori* infection [20,21]. Distinguishing physiological aging of the gastric mucosa from early pathological changes remains a challenge, especially in the absence of the *H. pylori* infection. Furthermore, commonly prescribed drugs such as proton pump inhibitors (PPIs) may induce changes in the gastric mucosa [22] that should be accounted for/evaluated.

Addressing the endoscopic and histological characteristics of the aging stomach is imperative since it may have several clinical implications. Firstly, age-related changes in the gastric mucosa may contribute to dyspepsia and the “anorexia of aging” [23]. Second, it has the potential to cause malabsorption, namely of iron, folate and, eventually, vitamin B12, increasing the risk of hematological disorders and a broad spectrum of neurological, cognitive and psychiatric manifestations [24,25,26]. Third, since incidence of gastric cancer (GC) rises progressively with age [27], the progression to chronic atrophic gastritis (CAG) and/or IM, a known risk factors for GC, should be accounted for, although the real risk is difficult to estimate because the prevalence of CAG in asymptomatic adults has not been clearly ascertained [28]. The scientific literature presents an important knowledge gap due to a lack of robust endoscopic and histological data comparing younger and older adults. It remains to be clarified if there is a distinct aging-associated phenotype in the gastric mucosa and how it may influence our pathology reports. Without knowing the differential processes associated with aging, the prevention, treatment and surveillance of digestive diseases may be inadequate.

In this study, we aimed to assess and compare potential clinical, endoscopic and histologic changes in the gastric mucosa associated with aging, and to explore whether aging is associated with distinct histological features or with a histological pattern.

## 2. Materials and Methods

### 2.1. Study Design

This was a single-center observational study conducted in a tertiary hospital. The present study was approved by the institutional ethics committee under authorization 71/2023. Due to the noninterventional nature of this study, standard clinical practice dictated patient management.

### 2.2. Patients

Consecutive younger (18–45 years) and older (≥70 years) adults undergoing elective upper endoscopy were recruited. Additional inclusion criteria included acceptance to participate in the study. For each patient enrolled, the following methodology was applied:

1. Prospective clinical and demographic database registry including age, gender, clinical indication for upper endoscopy and ongoing therapies with a special focus on antiplatelet, anticoagulant and PPIs. PPI therapy was defined as ongoing when drugs were suspended less than 7 days before endoscopy.
2. Upper endoscopy, under deep sedation, was performed according to several quality indicators, including the use of virtual chromoendoscopy, preprocedural simethicone and fundus retroflexion [29].
3. Endoscopic diagnoses were coded and collected in a predefined, standardized and searchable fashion. Antithrombotic therapy was managed according to international guidelines [30].
4. Two biopsy specimens were systematically obtained from the antrum, another two from the body and were compared for histological features. When endoscopic lesions (e.g., polyps, ulcer) were evident, apart from the respective lesion biopsies, random biopsies from the opposite wall of the antrum/body were collected for analysis.

All participants or their representatives signed an informed consent form.

Exclusion criteria in this study included:

1. Refusal to participate in the study.
2. Pregnancy.
3. Previously known gastric pathology (e.g., autoimmune gastritis, GC).
4. Recent *H. pylori* eradication (within 12 months).
5. Previous gastric surgery.
6. Crohn’s disease.
7. Chemoradiation and/or immunomodulatory therapy.
8. Technical impossibility to obtain gastric biopsies.

### 2.3. Gastric Biopsy Analysis

The gastric biopsies were evaluated by two pathologists, including one expert gastrointestinal pathologist, using optical microscopy. All biopsies were first examined with hematoxylin and eosin (H&E) and routinely submitted to Warthin–Starry (WS) silver staining for the detection of *Helicobacter pylori*. Immunohistochemistry (IHC) for *H. pylori* was reserved for cases with high clinical or histological suspicion in which both H&E and WS were negative, as well as for biopsies with advanced atrophy or intestinal metaplasia, or in patients with a prior eradication history. The infection was diagnosed by the visualization of characteristic curved organisms in the gastric mucosa. Uniform diagnostic criteria and standardized diagnostic codes were applied, and cases were reviewed in consensus meetings when necessary. All biopsies were assessed following the Updated Sydney System classification [31], as well as the Operative Link on Gastritis Assessment (OLGA) and Operative Link on Gastritis/Intestinal-Metaplasia Assessment (OLGIM) staging systems [32].

Chronic active gastritis was diagnosed in the presence of a mixed inflammatory infiltrate in the lamina propria and/or the in the surface of the foveolar epithelium, including lymphocytes, plasma cells and polymorphonuclear leukocytes, with no mucosal atrophy or IM. CAG was diagnosed by the loss of oxyntic glands. IM was identified by the presence of goblet cells within the gastric epithelium. Reactive gastropathy, resulting from long-term exposure to several substances capable of promoting gastric injury (e.g., bile reflux, medications), was identified based on combinations of foveolar hyperplasia, regenerative epithelial changes, edema/hyperemia of the lamina propria, erosions and smooth muscle proliferation [33]. PPI-related gastric changes comprise histopathological features that include oxyntic glandular hyperplasia, cystic dilation and increased glandular crowding [22]. Gastric mucosa was considered normal when no histopathological abnormalities were observed.

### 2.4. Statystical Analysis

Descriptive statistical analyses were performed using R (version 4.3.1) and Microsoft Excel (version 16.89.1, 2024). Normality was assessed using the Kolmogorov–Smirnov test. Continuous variables were summarized as mean ± standard deviation, and categorical variables as frequencies and percentages. Group comparisons employed the Chi-square test or Fisher’s exact test for categorical data, and Student’s t-test or Mann–Whitney U test for continuous variables.

For inferential analyses, penalized logistic regression with L2 regularization (Ridge regression) was the primary analytical framework, applied across five gastric pathology outcomes: chronic gastritis, intestinal metaplasia, CAG and PPI-related changes. An all-in model-building strategy was adopted, with a priori variable selection informed by clinical relevance and the prior literature. Two models were systematically evaluated for each outcome: a main-effects model including all predictors and an interaction model incorporating pre-specified age interactions (e.g., age group × PPI use; age group × H. pylori infection), reflecting hypotheses of age-dependent effects. Categorical predictors were encoded using reference coding: sex was binary (female = 0; male = 1), age group was dichotomized (18–45 years = 0; ≥70 years = 1), and the Charlson comorbidity index was categorized into 0 (reference), 1–2, 3–4, and ≥5. Antiplatelet therapy, anticoagulant therapy, PPI use, NSAID use and H. pylori infection were all entered as binary indicators.

Logistic regression was implemented in scikit-learn (version 1.3+) with hyperparameter optimization via LogisticRegressionCV. The regularization parameter (C) was selected through 5-fold stratified cross-validation over a grid of 50 logarithmically spaced values (10^−4^–10^4^), using the area under the receiver operating characteristic curve (AUC) as the performance metric. Interaction terms were retained if statistically significant (*p* < 0.05), with priority given to biological plausibility when highly significant (*p* < 0.001). Model parsimony was evaluated using the Akaike Information Criterion (AIC) and Bayesian Information Criterion (BIC). Variance Inflation Factors (VIFs) were calculated to assess multicollinearity, with values >5 considered concerning and >10 problematic; however, L2 regularization mitigated instability by shrinking correlated coefficients, even when extreme VIF values were present.

Internal validation was conducted with 1000 bootstrap resamples, repeating the full modeling procedure, including hyperparameter tuning, for each sample. Discrimination was assessed using apparent and optimism-corrected AUC, while calibration was evaluated with the Hosmer–Lemeshow test (five quantile-based bins; χ^2^ < 15 indicating adequate fit) and the Brier score. Optimism was defined as the difference between bootstrap and original sample performance, and corrected AUC was calculated as the apparent AUC minus mean optimism. Confidence intervals for coefficients and odds ratios (ORs) were obtained from the bootstrap distribution. For significant predictors, multiple effect measures were reported, including ORs with 95% confidence intervals, risk ratios calculated via the delta method and risk differences with binomial variance. Marginal absolute risks were derived by varying each predictor while fixing others at sample means, providing clinically interpretable risk estimates.

Although Firth’s bias correction was considered due to modest event rates (16–39%), L2 regularization yielded comparable bias reduction with greater computational efficiency. All analyses were implemented in Python 3.14.0. using scikit-learn for regression, NumPy and SciPy for statistical computations and custom functions for validation and effect measure calculations. A fixed random seed (42) ensured reproducibility, statistical significance was set at α = 0.05 and Bonferroni correction was applied when multiple comparisons were relevant. Forest plots were generated to visually represent odds ratios with 95% confidence intervals, displayed on a logarithmic scale where appropriate, highlighting clinically meaningful effect sizes.

## 3. Results

### 3.1. Baseline Clinical Characteristics

**Figure 1:**
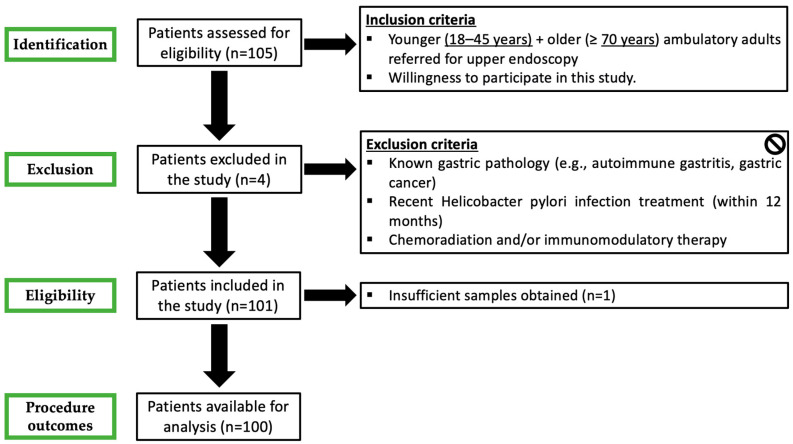
Patient flowchart in this study. One hundred and five patients were initially enrolled in the study and underwent upper endoscopy. Gastric biopsies were taken during the procedure for histological analysis. In total, 100 patients were available for complete analysis.

The patient flowchart of this study is detailed in Figure 1. One hundred and five patients were initially enrolled in the study and underwent upper endoscopy. Gastric biopsies were taken during the procedure for histological analysis. In total, 100 patients were available for complete analysis.

One hundred patients who fulfilled the inclusion criteria were included in the study (45 men/55 women), 50 with 18–45 years and 50 with ≥70 years. The clinical indication for upper endoscopy was dyspepsia for most patients of both groups, followed by peptic ulcer disease in younger adults and gastroesophageal reflux disease in older adults, the latter with a significant statistical difference (*p* = 0.019). The Charlson comorbidity index (CCI) was significantly higher in the elderly group (*p* < 0.001). Intake of nonsteroid anti-inflammatory drugs (NSAIDs), antiplatelets, anticoagulants and PPIs were more common in older adults. Time from symptom onset to endoscopy was similar between both study groups (4.5 ± 1.2 vs. 5.3 ± 1.7 months, *p* = 0.09).

The patients’ baseline characteristics are described in Table 1.

### 3.2. Gastric Endoscopic Lesions

At endoscopy examination, at least one gastric lesion was found in 50% of younger adults and 80% of older adults. Half of the patients with 18–45 years presented normal gastric mucosa at endoscopy, which contrasts with the elderly group, where erythematous gastropathy was the main endoscopic finding, followed by peptic ulcer disease. The prevalence of endoscopic lesions in the stomach was far more common in older adults (*p* = 0.003). Table 2 summarizes the distribution of gastric endoscopic lesions according to age group.

### 3.3. Gastric Histology

Histopathology examination differed between the two groups: normal gastric mucosa was more common in young adults (*n* = 22, 44%), followed by chronic (non-atrophic/metaplastic) gastritis (*n* = 19, 38%). On the other hand, chronic gastritis was the most common histologic finding in older adults (*n* = 28, 56%), followed by CAG/IM (*n* = 14, 28%). From the two younger patients with CAG/IM, those changes were located only in the antrum. In contrast, from the 14 older patients with CAG/IM, in three of them those changes were located only in the antrum, in two patients only in the body, and in the remaining nine patients in both the antrum and the body. Reactive gastritis did not differ between the two groups.

Overall, there is a decline in the prevalence of normal gastric mucosa in the older group. Moreover, 30% (*n* = 15) of elderly patients exhibited gastric changes commonly associated with chronic PPI therapy: oxyntic glandular hyperplasia, cystic dilation and increased glandular crowding. Global interobserver agreement (*Kappa*) between pathologists was almost perfect (0.92).

The prevalence of *H. pylori* in patients without PPI therapy was 32% (*n* = 16) in the younger group and 28% (*n* = 14) in the elderly group (*p* = 0.189).

Table 3 summarizes the distribution of histologic lesions according to age group.

**Figure 2:**
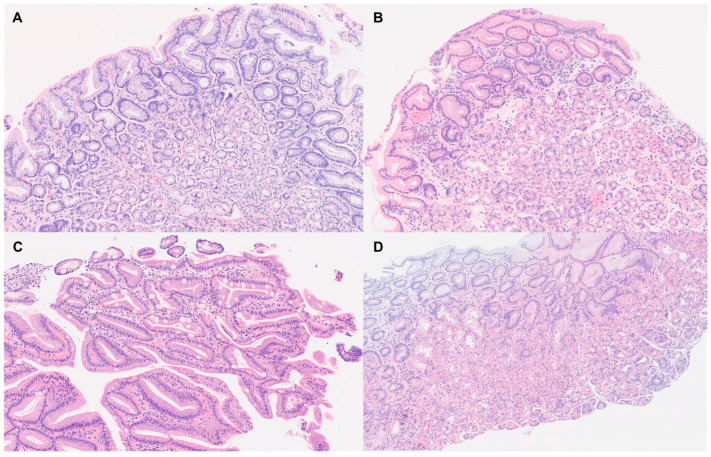
Histological findings: (**A**). Normal gastric mucosa: surface foveolar epithelial cells with mucin production, glands predominantly lined by mucous-secreting cells, scattered endocrine cells and occasional chief cells. The glandular and epithelial architecture is preserved (H&E, ×10). (**B**). Chronic (non-atrophic/metaplastic) gastritis: The lamina propria is expanded by a chronic inflammatory infiltrate composed predominantly of lymphocytes and plasma cells, with occasional lymphoid aggregates (H&E, ×10). (**C**). Chronic gastritis with intestinal metaplasia and atrophy: chronic inflammation within the lamina propria, with intestinal metaplasia and mild glandular atrophy (H&E, ×10). (**D**). Proton pump inhibitor-related gastric mucosal changes: Oxyntic glandular hyperplasia, cystic dilation and increased glandular crowding, commonly associated with proton pump inhibitors (H&E, ×10).

Figure 2 shows histological findings which differ between both groups.

After adjusting for confounders in the penalized logistic regression analyses, each histopathological outcome was shaped by a distinct set of predictors.

**Figure 3:**
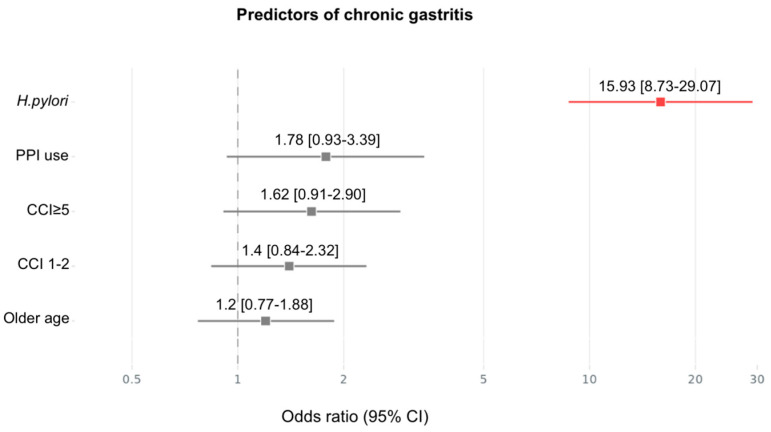
Forest plot illustrating the adjusted ORs and 95% CIs for predictors of chronic gastritis. *H. pylori* infection, PPI use, CCI ≥ 5 and older age reached statistical significance in the adjusted model. PPI—proton pump inhibitor; CCI—Charlson comorbidity index.

For chronic (non-atrophic/metaplastic) gastritis (Figure 3), risk was strongly elevated by *H. pylori* infection (OR = 15.93, 95% CI: 8.73–29.07), which emerged as the most powerful predictor in this model. PPI use (OR 1.78, 95% CI: 0.93–3.39), CCI ≥ 5 (OR = 1.62, 95% CI: 0.91–2.9) and older age (OR 1.2, 95% CI: 0.77–1.88) also contributed significantly to chronic gastritis risk.

**Figure 4:**
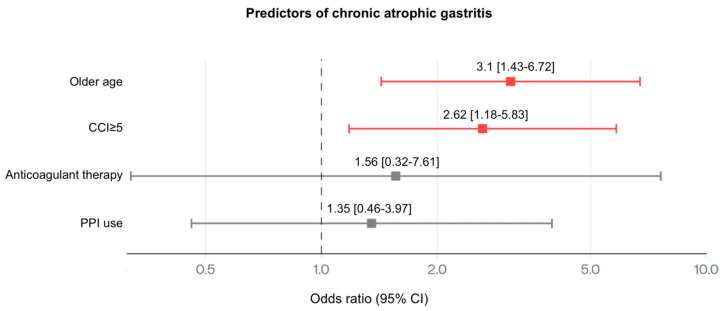
Forest plot illustrating the adjusted ORs and 95% CIs for predictors of chronic atrophic gastritis. Older age, CCI ≥ 5, anticoagulant therapy and PPI use were significantly associated with higher odds of chronic atrophic gastritis. PPI—proton pump inhibitor; CCI—Charlson comorbidity index.

In the case of CAG (Figure 4), older age (OR 3.1, 95% CI: 1.43–6.72) and CCI ≥ 5 (OR 2.62, 95% CI: 1.18–5.83) were identified as independent predictors using logistic regression with L2 regularization. Anticoagulant therapy (OR 1.56, 95% CI: 0.32–7.61) and PPI therapy (OR 1.35, 95% CI: 0.46–3.97) were also linked to substantially increased risk.

**Figure 5:**
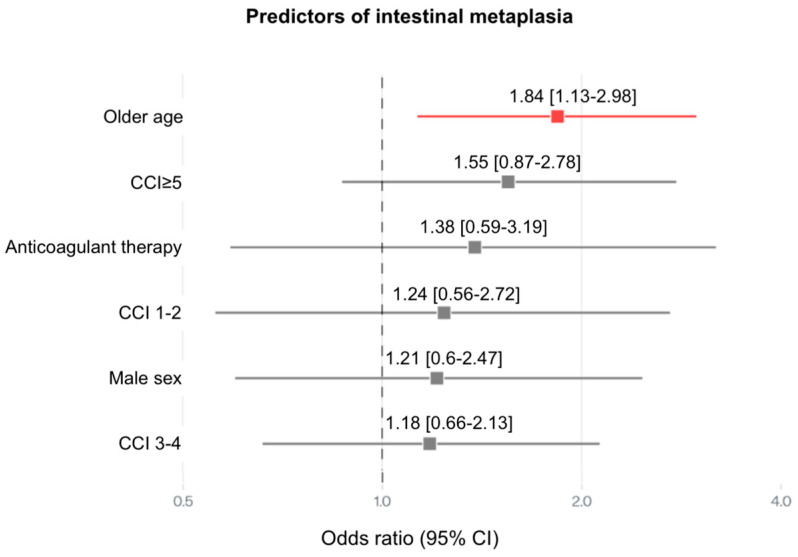
Forest plot illustrating the adjusted ORs and 95% CIs for predictors of intestinal metaplasia. Significant associations were observed with older age, CCI, anticoagulant therapy and male sex, all contributing to elevated odds. CCI—Charlson comorbidity index.

Regarding IM (Figure 5), older age (OR 1.84, 95% CI: 1.13–2.98) and age/PPI interaction (OR 1.83, 95% CI: 1.03–3.22) were the primary independent predictors. CCI ≥ 5 (OR 1.55, 95% CI: 0.87–2.78), anticoagulant therapy (OR 1.30, 95% CI: 0.59–3.19) and male sex (OR 1.21, 95% CI: 0.60–2.47) also contributed significantly to increased risk of IM.

For PPI-related gastric changes, apart from PPI therapy (OR 5.42, 95% CI: 2.53–11.61), age/PPI interaction (OR 3.54, 95% CI: 1.85–6.78) was linked to substantially increased risk.

Full models’ performance indexes, predictors and interactions are included in Table S1 (supplementary file).

## 4. Discussion

This study provides a comparative clinical, endoscopic and histological analysis and updated data of the gastric mucosa in younger and older patients undergoing upper endoscopy in the routine practice of a gastroenterology department. Dyspepsia, gastroesophageal reflux disease and peptic ulcer disease as the most frequent indications for upper endoscopy are in line with other European centers [34]. Patients with prior history of gastric pathology, including autoimmune gastritis, a form of metaplastic/atrophic gastritis, were a priori excluded to avoid the overestimation of gastric findings on both study groups.

Prior evidence suggests that the gastric mucosa undergoes progressive structural and functional changes with age [35]. However, an important number of studies preceded the recognition of *H. pylori* infection and its potential effect on the gastric mucosa. Our findings demonstrate that aging may be associated with distinct histological changes, namely chronic inflammation, CAG and IM, in the absence of overt gastric disease. These results suggest that aging may be associated with a specific gastric mucosal phenotype, with possible implications for both pathophysiological understanding and clinical interpretation. The histopathological examination of both study groups clearly revealed a predominance of changes in the older group, changes that are traditionally interpreted as biomarkers of increased GC risk [29]. Furthermore, most of those changes were stage III/IV of the OLGA/OLGIM systems, associated with an increased risk of GC [32]. This raises an important consideration: age-related changes in the gastric mucosa present as early stages of the Correa cascade. In future longitudinal and prospective studies, this should be accounted for regarding clinical risk stratification for GC [36,37,38].

The increased prevalence of chronic gastritis observed in older patients may reflect a phenomenon of chronic low-grade inflammation characteristic of aging, often termed “*inflammaging*” [39]. This process involves a sustained, systemic inflammatory state driven by immunosenescence, epithelial stress and microbial shifts, which may affect tissue homeostasis in the gastrointestinal tract [40]. While *H. pylori* remains a central etiological factor in chronic gastritis and carcinogenesis, our data raises the hypothesis that aging itself may independently promote an inflammatory gastric microenvironment, along with other environmental factors [41,42].

PPIs are among the most prescribed drugs to elderly patients for the treatment of heartburn, gastroesophageal reflux disease, peptic ulcer disease and *H. pylori* infection [43]. Moreover, PPIs are also commonly prescribed for “gastric protection” in patients undergoing polypharmacy. A recent review highlighted some potential side effects associated with long-term use: osteoporotic fractures, *Clostridioides difficile* infection, community-acquired pneumonia, vitamin B12 deficiency and kidney disease [44,45]. Moreover, this antisecretory therapy, when used long-term, is associated with several endoscopic (gastric polyps, multiple white and flat mucosal elevations, cobblestone-like mucosa and black spots) and histologic findings (oxyntic glandular hyperplasia, cystic dilation, increased glandular crowding, parietal cell protrusions and foveolar epithelial hyperplasia) [46]. Although the clinical importance of these mucosal changes is not consensual [22], on multivariate analysis, PPI use was a significant predictor of CAG, and age/PPI interaction was also a predictor of IM. Furthermore, older age was also associated with the risk of PPI-related gastric changes, acting as a possible cofactor for these histological changes. A recent cohort study pointed out a 45% increased risk of gastric cancer compared with the use of histamine-2 receptor antagonists, associated with cumulative duration of use, cumulative omeprazole equivalents and time since treatment initiation [47]. Our finding that PPI use correlates with CAG may result from reverse causality, given the cross-sectional design of this study. However, Calabrese et al. [48] found an association between PPI use and advanced stages of CAG and IM, in line with other studies [49]. Moreover, it is known that chronic gastritis, IM and CAG are conditions associated with the inappropriate use of PPIs and those patients are more likely to receive PPIs in clinical practice [50]. In patients without valid indications, PPI therapy should be discontinued. When indicated for long-term therapy, the lowest effective dose should be used, and its need should be periodically reassessed [48]. Nevertheless, due to the uncertain clinical impact of these histological changes and for evaluating causality, a longitudinal and prospective study including indication, molecule/dose and cumulative duration would better answer these unsolved questions.

Stratified analyses showed that the coexistence of advanced age and *H. pylori* infection further exacerbates mucosal damage, consistent with earlier studies showing the synergistic effects between microbial insult and host aging [51,52]. However, there remains a significant gap in understanding how aging influences these pathological processes [53]. Even among *H. pylori*-negative patients, older age was still associated with atrophic and metaplastic changes, supporting the hypothesis that aging contributes to the structural remodeling of the gastric mucosa. There are several pathological mechanisms that may account for this hypothesis, aging may (1) alter the host’s immune response to *H. pylori* [12], (2) impair epithelial repair mechanisms [11] and/or (3) lead to the progressive depletion of gastric stem cell populations [54]. We did not assess the molecular markers of stem cell turnover or regenerative capacity, but such studies could further clarify the mechanisms underpinning these age-associated changes. In fact, emerging studies using animal models have shown similar age-related alterations in gastric gland architecture, stem cell exhaustion and decreased parietal cell numbers—changes which may contribute to the observed histological patterns in human aging [4,55]. Whether these changes are adaptive, degenerative or pre-neoplastic remains an open question.

The recognition of a possible aging stomach phenotype has several important clinical implications. Firstly, our findings showed an increased prevalence of chronic gastritis, IM and CAG in older adults, which may result from decades of cumulative gastric injury by previously eliminating *H. pylori* and multiple aggressive factors other than *H. pylori*. In this regard, a significant number of elderly patients may already meet histological thresholds for surveillance [29]. This could either lead to over-surveillance or missed opportunities for early GC detection, depending on how age-related changes are interpreted. It remains to be seen if these non-*H. pylori* associated changes have the same risk of GC, which may underscore the need for the age-adjusted interpretation of gastric histology, particularly in asymptomatic elderly patients. Indeed, findings such as mild atrophy or focal intestinal metaplasia in older citizens may not necessarily indicate high risk for neoplastic progression in this context. Conversely, understanding the cumulative impact of aging and environmental exposure may refine risk prediction models for GC. Indeed, Lahner et al. [56] challenged the traditional concept of GC typically arising from multifocal CAG in the context of *H. pylori* infection. Their data showed that body-restricted CAG might be associated with gastric dysplasia and GC more frequently than expected. In line with our results, these findings suggest endoscopic–histological surveillance for patients with gastric body CAG, irrespective of extension and staging. Also, the aforementioned structural changes may account for the age-related decline in gastric secretion, with an important clinical impact, as recently reviewed by our *Aging Lab* team [8]. Future guidelines should consider the physiological baseline of the aging gastric mucosa.

While few autopsy [28], retrospective [57] and prospective studies [58] have hinted at age-related changes in the stomach, we have systematically compared young and elderly patients using a standardized biopsy protocol routinely performed at our center and histological assessment. Francesco et al. [59] showed an increased prevalence of gastric histological lesions in patients > 50 years. However, the age cut off may not reflect actual worldwide demographics and it seems prudent to use a higher one, such as the commonly cited chronological age of 65 years old that usually defines an older person [3]. Our study adds a comparative dimension and excludes confounders such as recent *H. pylori* eradication or chronic PPI use.

*H. pylori* infection remains a very important infection worldwide. Although global improvements in healthcare in developed countries have led to a decline in its prevalence among younger groups, the infection remains common in older adults [12]. In our study, prevalence did not differ between groups and was lower compared with other studies [60,61]. Indeed, the prevalence of *H. pylori* infection among those aged ≥75 years decreases markedly, possibly due to widespread antibiotic and PPI use, as well as resulting from the higher prevalence of CAG [62].

The strengths of this study include standardized biopsy sampling and age-stratified analysis. Furthermore, our observational study represents a comprehensive analysis of younger and older patients referred to upper endoscopy in a real-world clinical setting. Importantly, biopsies from the antrum and body were assessed, allowing for a detailed regional comparison of mucosal changes with age. However, several limitations should be acknowledged. The study sample included patients with a clinical indication for upper endoscopy, introducing a selection bias. Gastrointestinal/alarm symptoms correlate with endoscopic lesions, which increases their prevalence in both study groups [54]. However, it would be unethical to perform endoscopy without a clinical rationale. Population-based screening programs for GC and precancerous conditions are not implemented worldwide except in high-risk populations according to MAPS III guidelines [29]. The study is observational in nature, and although we excluded patients with known gastric pathology, unrecognized subclinical processes cannot be fully ruled out. Histological analysis was performed in both study groups, and the findings were compared, but this evaluation was not blinded. This may increase the risk of biased pathological reports according to patient clinical information. However, in a real-world study this allows the pathologist to have a comprehensive understanding of the patient and identify subtle changes that might otherwise be missed. Furthermore, the biopsies were analyzed by at least two operators using the same standardized protocol to avoid interobserver variability, reaching an almost perfect agreement (k = 0.92). Functional assessments of acid secretion, pepsinogen levels or gastric hormone profiles were not performed, limiting correlation between histological and physiological aging. Also, no molecular or microbiome data were collected, which could have provided further insight into mucosal remodeling mechanisms. Furthermore, our sample size of 100 patients may reduce the generalizability of our results, as well as the statistical power for subgroup analyses. However, the findings of this study already show a trend towards chronic gastritis, IM and CAG in the elderly, calling for a longitudinal study with a larger sample size.

Future studies should aim to integrate molecular markers of aging, such as senescence-associated β-galactosidase, telomere shortening or stem cell integrity, into histopathological analyses. Moreover, since the prevalence of autoimmune gastritis increases with age [63] and is an important differential diagnosis of IM and CAG in the elderly [64], this should also be addressed to ascertain its prevalence and its possible association with gastric aging. The role of the microbiome in modulating age-related inflammation and GC development is increasingly recognized [65,66] and warrants further investigation. Lastly, the use of digital pathology and artificial intelligence-based scoring could enhance reproducibility and detect subtle morphologic changes not easily identified by conventional microscopy.

## 5. Conclusions

The present study suggests the presence of distinct histological features in the gastric mucosa of older individuals, characterized by chronic inflammation, increased glandular atrophy and IM, even in the absence of *H. pylori* infection or overt disorders. Resulting from several aging-related pathophysiological processes and decades of cumulative gastric injury, aging may give rise to an older gastric mucosa phenotype, which could underlie an increased risk for several disorders including GC.

Our findings underscore the importance of the age-adjusted interpretation of gastric histology and support for future prospective research into the mechanisms of gastric mucosal aging. Integrating histological, molecular and functional data may become the key to guiding appropriate clinical management in elderly patients.

## Figures and Tables

**Table 1:** Baseline patient characteristics. NSAIDs—non-steroid anti-inflammatory drugs. SD—standard deviation.

Variables		18–45 Years (*n* = 50)	≥70 Years (*n* = 50)	*p* Value
Age (SD)		33.6 ± 8.1 years	76.2 ± 4.1 years	
Gender	Male	23 (46%)	22 (44%)	0.421
	Female	27 (54%)	28 (56%)	
Charlson Comorbidity index		0.2 ± 0.4	4.8 ± 1.6	<0.001
Ambulatory therapy	NSAID	1 (2%)	8 (16%)	<0.001
	Antiplatelets	3 (6%)	16 (32%)	<0.001
	Anticoagulants	2 (4%)	8 (16%)	0.023
	Proton pump inhibitors	9 (18%)	21 (42%)	<0.001
Laboratory evaluation	Hemoglobin (SD)	13.6 ± 1.3	12.9 ± 1.7	0.044
	Iron (SD)	82.3 ± 40.0	73.1 ± 32.6	0.377
	Folic acid (SD)	5.4 ± 4.3	5.1 ± 4.8	0.234
	Vitamin B12 (SD)	601 ± 87.6	549 ± 69.5	0.565
Clinical indication for upper endoscopy	Dyspepsia	23 (46%)	17 (34%)	0.079
	Gastroesophageal reflux disease	5 (10%)	10 (20%)	0.019
	Peptic ulcer disease	10 (20%)	9 (18%)	0.401
	Dysphagia	4 (8%)	3 (6%)	0.349
	Radiological findings	3 (6%)	2 (4%)	0.325
	Gastric polyp	2 (4%)	4 (8.0%)	0.202
	Diarrhea	2 (4%)	3 (6%)	0.325
	Iron deficiency anemia	1 (2%)	2 (4%)	0.281

**Table 2:** Distribution of lesions detected at upper endoscopy according to age group. Bold data refers to statistical differences.

Endoscopic Finding		18–45 Years (*n* = 50)	≥70 Years (*n* = 50)	*p* Value
**Normal mucosa**		**25 (50%)**	**10 (20%)**	<0.001
Erythema		17 (34%)	15 (30%)	0.336
Erosions		5 (10%)	8 (16%)	0.189
Peptic ulcer disease	**Gastric ulcer**	**1 (2%)**	**4 (6%)**	0.004
	**Duodenal ulcer**	**0 (0%)**	**6 (12%)**	
**Gastric polyps**		**2 (4%)**	**7 (14%)**	0.041

**Table 3:** Distribution of histological findings detected according to age group. PPI—proton pump inhibitor. *H. pylori*—*Helicobacter pylori*. OLGIM—Operative Link on Gastritis/Intestinal-Metaplasia Assessment. OLGA—Operative Link on Gastritis Assessment. Bold data refers to statistical differences.

Histologic Finding		18–45 Years (*n* = 50)	≥70 Years (*n* = 50)	*p* Value
**Normal mucosa**		**22 (44%)**	**2 (4%)**	<0.001
Reactive gastritis		7 (14%)	5 (10%)	0.271
Reactive gastritis *H.pylori* +		3 (6%)	0 (0%)	0.0910
**Chronic gastritis**		**19 (38%)**	**28 (56%)**	0.004
Chronic gastritis *H.pylori* +		11 (22%)	10 (20%)	0.0681
**Intestinal metaplasia**	**OLGIM I**	**2 (4%)**	**2 (4%)**	<0.001
	**OLGIM II**	**0 (0%)**	**3 (6%)**	
	**OLGIM III**	**0 (0%)**	**5 (10%)**	
	**OLGIM IV**	**0 (0%)**	**4 (8%)**	
Intestinal metaplasia *H.pylori* +		1 (2%)	2 (4%)	0.113
**Chronic atrophic gastritis**	**OLGA I**	**2 (4%)**	**3 (6%)**	<0.001
	**OLGA II**	**0 (0%)**	**3 (6%)**	
	**OLGA III**	**0 (0%)**	**4 (8%)**	
	**OLGA IV**	**0 (0%)**	**4 (8%)**	
Chronic atrophic gastritis *H.pylori* +		1 (2%)	2 (4%)	0.113
**PPI-related gastric changes**		**2 (4%)**	**15 (30%)**	<0.001
*H.pylori* +		0 (0%)	0 (0%)	

## Data Availability

All the data analyzed during this study are included in this article. Further inquiries can be directed to the corresponding author.

## Supplementary Materials

The following supporting information can be downloaded at: https://www.mdpi.com/article/10.3390/medsci13040224/s1, Table S1: Full models’ performance indexes, predictors and interactions.

## Abbreviations

The following abbreviations are used in this manuscript:

CCI	*Charlson comorbidity index*
CAG	Chronic atrophic gastritis
GC	Gastric cancer
H. pylori	Helicobacter pylori
IM	Intestinal metaplasia
PPI	Proton pump inhibitor
