# Supplementary material for: Clinical, Endoscopic and Histologic Differences in Gastric Mucosa Between Younger and Older Adults: An Observational Study on the Aging Stomach
Source: Med Sci (Basel). 2025 Oct 8;13(4):224. doi: 10.3390/medsci13040224 (PMC12550935; doi:10.3390/medsci13040224)
Supplement: Supplementary file 1 [file medsci-13-00224-s001.zip › medsci-3852182-supplementary.pdf]

Table S1: Models' performance indexes, predictors and interactions.

Outcome	Apparent AUC	Corrected AUC	Bootstrap 95% CI for AUC	Hosmer-Lemeshow χ^2	Brier Score	Bootstrap internal validation (n=1000) optimism	Significant predictors ($p < 0.05$)	Age group interactions
Chronic Gastritis	0.932	0.909	0.885 - 0.982	7.287 (good calibration)	0.108	0.024 (excellent)	**H. pylori infection**<br>• **OR**: 15.93 (95% CI: 8.73-29.07), p < 0.001<br>• **Risk Ratio**: 5.14 (95% CI: 3.11-8.50)<br>• **Risk Difference**: +0.748 (95% CI: 0.618-0.878)<br>**Marginal absolute risk**: 82.2% vs 22.5%	None
Chronic atrophic gastritis	0.806	0.726	0.730 - 0.940	1.035 (excellent calibration)	0.109	0.081 (moderate)	**Age ≥70 years**<br>• **OR**: 3.10 (95% CI: 1.43-6.72), p = 0.004<br>• **Risk Ratio**: 7.00 (95% CI: 1.68-29.22)<br>• **Risk Difference**: +0.240 (95% CI: 0.104-0.376)<br>• **Marginal absolute risk**: 19.8% vs 7.4%	None

							**Charlson comorbidity index ≥5**<br>• **OR**: 2.62 (95% CI: 1.18-5.83), p = 0.018<br>• **Risk Ratio**: 7.44 (95% CI: 1.74-31.82)<br>**Risk Difference**: +0.300 (95% CI: 0.106-0.493)	
Intestinal Metaplasia	0.796	0.691	0.748 - 0.930	2.441 (excellent calibration)	0.115	0.105 (substantial)	**Age ≥70 years**<br>• **OR**: 1.84 (95% CI: 1.13-2.98), p = 0.014<br>• **Risk Ratio**: 7.00 (95% CI: 1.68-29.22)<br>• **Risk Difference**: +0.240 (95% CI: 0.104-0.376)<br>• **Marginal absolute risk**: 21.4% vs 12.9%<br>**Age × PPI Interaction**<br>• **OR**: 1.83 (95% CI: 1.03-3.22), p = 0.038<br>• **Interpretation**: PPI effect is significantly modified by age	PPI (p=0.038)
PPI-related gastric changes	0.964	0.950	0.939 - 0.995	4.045 (excellent calibration)	0.062	0.014 (exceptional)	**PPI use**<br>• **OR**: 5.42 (95% CI: 2.53-11.61), p < 0.001	PPI (p<0.001)

							• **Risk Ratio**: Not calculable (all PPI-related changes occurred in PPI users)<br>• **Risk Difference**: +0.486 (95% CI: 0.320-0.651)<br>**Age × PPI interaction (amplifies PPI risk)**<br>• **OR**: 3.54 (95% CI: 1.85-6.78), p < 0.001	
